# Design, Synthesis, and Evaluation of Doxifluridine Derivatives as Nitroreductase-Responsive Anticancer Prodrugs

**DOI:** 10.3390/molecules29215077

**Published:** 2024-10-27

**Authors:** Xinmeng Zhang, Taimin Dong, Xu Li, Changjie Xu, Fanghui Chen, Shiben Wang, Xuekun Wang

**Affiliations:** National Key Laboratory of Macromolecular Drug Development and Manufacturing, School of Pharmaceutical Sciences and Food Engineering, Liaocheng University, 1 Hunan Street, Liaocheng 252059, China; 18241016451@163.com (X.Z.); 17616455575@163.com (T.D.); lx221024@126.com (X.L.); 13306359838@163.com (C.X.); chenfanghui@lcu.edu.cn (F.C.)

**Keywords:** prodrug, doxifluridine derivatives, nitroreductase, tumor microenvironment

## Abstract

Antimetabolite antitumor drugs interfere with nucleic acid and DNA synthesis, causing cancer cell death. However, they also affect rapidly dividing normal cells and cause serious side effects. Doxifluridine (5′-deoxy-5-fluorouridine [5′-DFUR]), a 5-fluorouracil (5-FU) prodrug converted to 5-FU by thymidine phosphorylase (TP), exerts antitumor effects. Since TP is distributed in tumor and normal tissues, 5′-DFUR features side effects. Here we designed a series of novel 5′-DFUR derivatives based on high nitroreductase (NTR) levels in the hypoxic microenvironment of tumor tissues by introducing nitro-containing moieties into the 5′-DFUR structure. These derivatives exert their antitumor effects by producing 5-FU under the dual action of TP and NTR in the tumor microenvironment. The derivatives were synthesized and their stability, release, and cytotoxicity evaluated in vitro and antitumor activity evaluated in vivo. Compound **2c**, featuring nitrofuran fragments, was stable in phosphate-buffered saline and plasma at different pH values and reduced rapidly in the presence of NTR. The in vitro cytotoxicity evaluation indicated that compound **2c** showed excellent selectivity in the MCF-7 and HT29 cell lines. Moreover, it exhibited antitumor effects comparable to those of 5′-DFUR in vivo without significant toxic side effects. These results suggest that compound **2c** is a promising antitumor prodrug.

## 1. Introduction

5-Fluorouracil (5-FU), a uracil mimetic with a fluorine atom at the C-5 position instead of hydrogen, is an anti-metabolic chemotherapeutic agent, which is used to the treatment of colorectal, breast, stomach, esophageal and head/neck cancers [1]. The mechanism of 5-FU cytotoxicity has been attributed to the misincorporation of 5-fluoro-2′-deoxyuridine 5′-triphosphate into DNA instead of thymidine triphosphate or its conversion to 5-fluorouridine 5′-triphosphate, which competes with uridine triphosphate during RNA synthesis [2]. 5-FU can inhibit not only the growth of tumor cells but also the proliferation of rapidly dividing normal cells, causing a variety of undesirable side effects such as gastrointestinal distress, myelosuppression, and cardiotoxicity [3]. Based on the structure and mechanism of action of 5-FU, prodrugs of 5-FU were developed to reduce toxicity, extend the duration of action, and increase tumour selectivity, such as capecitabine, tegafur, carmofur and doxifluridine [4]. Among them, doxifluridine (5′-deoxy-5-fluorouridine [5′-DFUR]) is converted to 5-FU by the thymidine phosphorylase (TP) enzyme, which is abundant in tumour tissues. 5′-DFUR is commonly used to treat several malignant cancers, including gastrointestinal and breast, and has similar indications to 5-FU [5]. Compared to 5-FU, 5′-DFUR has a similar spectrum of activity and higher therapeutic index; however, side effects have been observed during therapy, such as gastrointestinal reactions, liver and kidney injury, bone marrow suppression, and neurotoxicity [6,7]. This may be because TP is distributed in tumors and the major organs, including the liver and small intestine. The 5-FU generated in the liver and small intestine is redistributed via the systemic circulation to tissues that lack phosphorylases, which is likely to contribute to these side effects [8,9]. Several tumor microenvironment-responsive drug delivery systems have been developed to reduce the side effects of doxifluridine [10,11]. Therefore, the selective metabolism of 5′-DFUR and release of 5-FU in tumor tissues can effectively reduce the side effects of antimetabolites.

**Figure 1:**
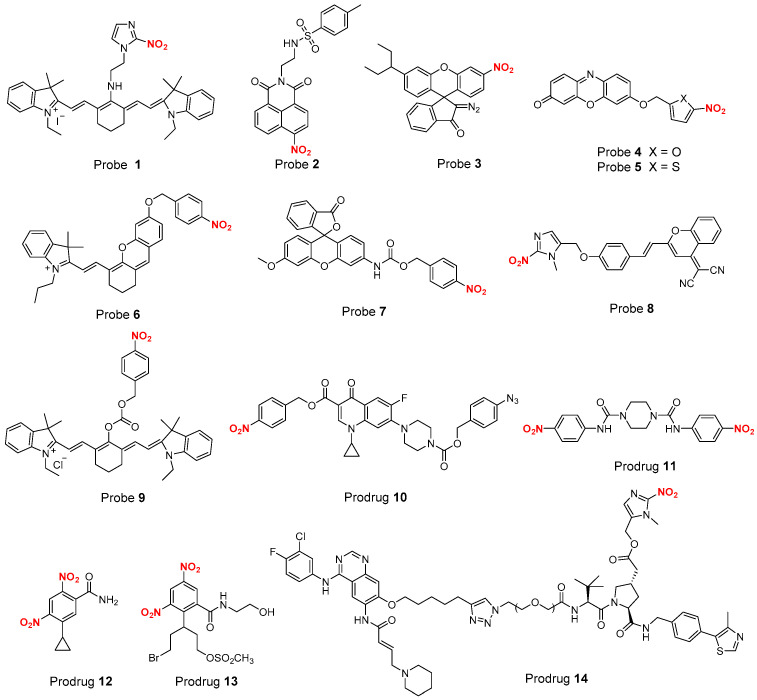
Representative fluorescent probes and prodrugs developed based on nitroreductase.

The tumor microenvironment (TME), a complex milieu involved in tumor development, comprises tumor epithelial cells, immune cells, surrounding blood vessels, fibroblasts, extracellular stroma, and various signaling molecules [12]. Within a rapidly proliferating tumor, the characteristics of the TME are significantly different from those of normal tissues, with weakly acidic interstitial cells [13], hypoxia [14], interstitial hypertension [15,16,17], and inflammatory reactivity [18]. Tumor hypoxia is believed to be caused, in part, by the relatively slow growth of blood vessels that cannot keep up with the demand of fast-growing tumor cells, resulting in the collection of hypoxic tumor cells in the vascularized regions of the tumor [19]. Tumor hypoxia affects tumor structure, function, and spread and plays a critical role in cancer aggression, invasiveness, and treatment resistance [20,21,22]. In hypoxic tumor cells, the expression of enzymes, such as nitroreductase (NTR), hexokinase II [23] and azoreductase [24], is increased. NTR is a flavin-dependent enzyme that can reduce nitroaromatic compounds or nitroheterocyclic derivatives to hydroxylamines or amines, with the assistance of coenzymes nicotinamide adenine dinucleotide (NADH) or nicotinamide adenine dinucleotide phosphate (NADPH) [25]. Over the past few decades, NTR has been identified as a biomarker for hypoxia and become an important target for tumor diagnosis and treatment. Owing to its physiological significance, numerous analytical methods have been developed to qualitatively and quantitatively detect NTR in tumor cells, and numerous prodrugs have been developed to treat tumors based on the high levels of NTR in tumor cells (Figure 1) [26,27,28,29,30,31,32,33,34,35].

**Figure 2:**
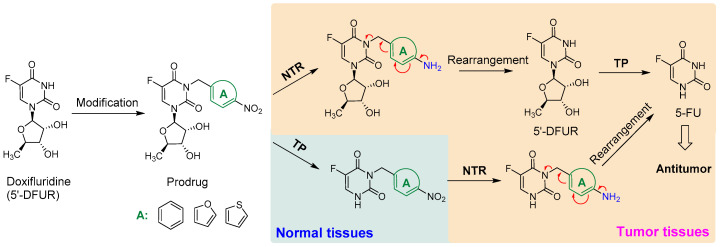
Design strategies and metabolic processes of nitroreductase-responsive prodrugs.

In this study, to reduce the side effects of 5′-DFUR, a series of novel 5′-DFUR derivatives was designed by introducing nitro-containing moieties into the structure of 5′-DFUR based on the higher NTR level in the TME. The designed derivatives were distributed to tumor and normal tissues. The derivatives distributed to tumor tissues produces antitumor effects after metabolizing and releasing 5-FU under the dual action of high concentrations of TP and NTR within tumor tissues. The derivatives distributed in the normal tissues is metabolized to produce a nitro derivative under the action of TP. This nitro derivative is redistributed to the tumor tissue and metabolized to release 5-FU under the action of NTR in the tumor tissue to exert antitumor activity. These derivatives reduced the release of 5-FU in normal tissues, effectively reducing the side effects of 5-FU (Figure 2). The designed derivatives were chemically synthesized and evaluated for their stability, release ability, cytotoxicity in vitro, and antitumor activity in vivo. The results showed that compound **2c** excellently released 5′-DFUR under the action of NTR in vitro but did not exhibit high cytotoxicity in vitro in the presence or absence of NTR. The in vivo evaluation showed that compound **2c** had comparable antitumor effects to those of 5′-DFUR, but as the body weights of the mice were higher than those of the 5′-DFUR group, fewer side effects were noted.

## 2. Results and Discussion

### 2.1. Chemistry

**Scheme 1:**
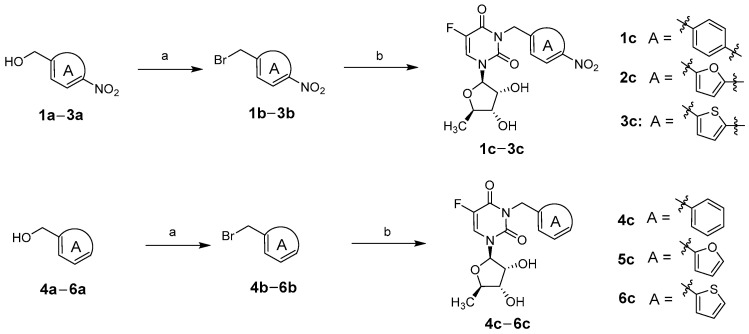
Synthesis of nitroreductase-responsive 5′-DFUR derivatives. Reagents and conditions: (**a**) PBr_3_, CH_2_Cl_2_, 0 °C, 1 h; (**b**) 5′-deoxy-5-fluorouridine, K_2_CO_3_, *N*,*N*-dimethylformamide, 25 °C, 12 h.

Target compounds **1c**–**6c** were obtained according to the synthetic route summarized in Scheme 1. The hydroxyl groups of starting materials 4-nitrobenzyl alcohol (**1a**), 5-nitrofurfuryl alcohol (**2a**), or (5-nitro-thiophen-2-yl)-methanol (**3a**) were replaced by phosphorus tribromide at 0 °C to obtain the corresponding bromine substitutes **1b**–**3b** as previously reported [36]. The treatment of bromine substitutes **1b**–**3b** with a molar equivalent of 5′-DFUR in the presence potassium carbonate in *N*,*N*-dimethylformamide at room temperature gave the target compounds **1c**–**3c**. To illustrate the important role of nitro groups in the design of the NTR-responsive prodrugs, we synthesized control compounds **4c**–**6c** without nitro-containing moieties. The synthetic method for the target compounds **4c**–**6c** was the same as that for **1c**–**3c**, using benzyl alcohol (**4a**), furfuryl alcohol (**5a**), or 2-thiophenemethanol (**6a**) as the starting materials. The structures of the target compounds **1c**–**6c** were confirmed by proton nuclear magnetic resonance (^1^H-NMR), carbon-13 nuclear magnetic resonance (^13^C-NMR), and high-resolution mass spectrometry (HRMS).

### 2.2. Biological Evaluation

#### 2.2.1. Stability Evaluation of Target Compounds in Phosphate-Buffered Saline Versus Plasma

**Figure 3:**
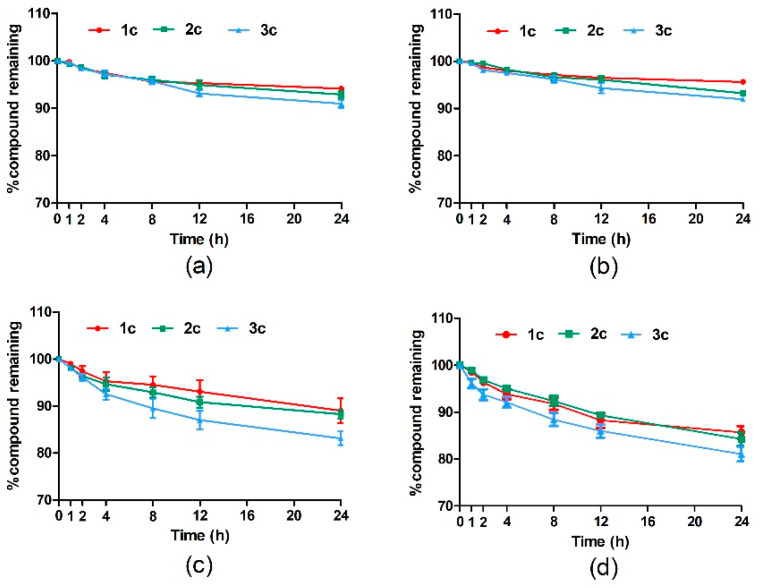
Stability of compounds **1c**–**3c** in different buffers and plasma: (**a**) phosphate-buffered saline (pH = 6.5); (**b**) phosphate-buffered saline (pH = 7.4); (**c**) mouse plasma; (**d**) rat plasma; All values were expressed as mean ± SEM (*n* = 3).

The stability of the target compounds was tested first, and excellent stability was essential for physiological function evaluation. Phosphate-buffered saline (PBS) of different pH values was used to evaluate the stability of the target compounds in a weakly acidic tumor microenvironment (pH 6.5) versus a weakly alkaline normal physiological environment (pH 7.4). All target compounds had superior stability in PBS (pH 6.5 and pH 7.4), and their concentrations were still >90% of the initial concentrations after 24 h, indicating a negligible effect of the difference in pH between normal and tumor tissues on their stability (Figure 3a,b). The stability of the target compounds in mouse and rat plasma was excellent. The concentrations of all target compounds remained at >80% of the initial concentrations after 24 h, with the concentrations of compounds **1c, 2c,** and **3c** being 89%, 88%, and 83% in mouse plasma and 86%, 84%, and 81% in rat plasma, respectively (Figure 3c,d). Compounds **4c**, **5c,** and **6c**, without nitro-containing moieties, were similarly stable in PBS at different pH values and plasma (Figure S1). These results indicate that the target compounds were not metabolized in the plasma to generate active products that cause side effects after their administration.

#### 2.2.2. In Vitro Release Evaluation of Target Compounds

**Figure 4:**
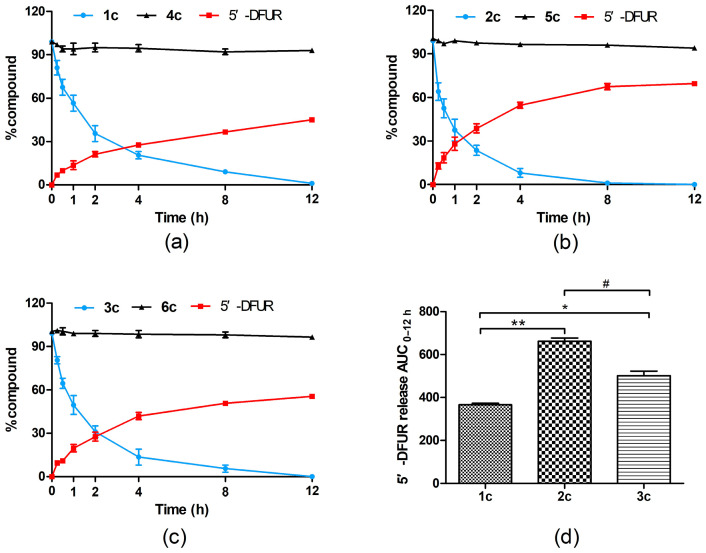
Percent of compounds remaining and 5′-DFUR releasing in 12 h of incubation with NTR in the presence of NADH. (**a**) compound **1c** and **4c**; (**b**) compound **2c** and **5c**; (**c**) compound **3c** and **6c**; (**d**) area under the curve (AUC_0–12h_) of the relative amount of released 5′-DFUR. 5′-DFUR, 5′-deoxy-5-fluorouridine. All values were expressed as mean ± SEM (*n* = 3) * *p* ≤ 0.05 compound **3c** compared with compound **1c** by Student’s *t* test; ** *p* ≤ 0.01 compound **2c** compared with compound **1c** by Student’s *t* test; ^#^ *p* ≤ 0.05 compound **2c** compared with compound **3c** by Student’s *t* test.

NTR levels are reportedly much higher in hypoxic tumors than in normal tissues. To investigate the mechanism of activation of the derivatives and the amount of 5′-DFUR released, we treated the target compounds with NTR extracted from *Escherichia coli* to determine whether they provided the desired active agent as expected. The test compounds (50 µM, 1% dimethyl sulfoxide [DMSO]) were dissolved in PBS buffer at pH 7.4 and incubated with NTR (50 µg/mL) and NADH (2 mM) at 37 °C, and the concentrations of the derivatives and 5′-DFUR during the bioreduction were detected by high-performance liquid chromatography (HPLC). The designed target compounds **1c**–**3c** could be metabolized to form 5′-DFUR in the presence of NTR, while the nitro-free compounds **4c**–**6c** could not be metabolized to form 5′-DFUR. Compound **1c** had a half-life of 1.3 h and was completely degraded after 12 h, while 45.1% of 5′-DFUR was released. The metabolic rate of compound **2c** was the highest, with a half-life of 0.6 h. It was completely degraded after 4 h, and 69.6% of 5′-DFUR was released. The half-life of compound **3c** was 1.0 h, and it was completely degraded after 8 h; 55.6% of the 5′-DFUR was released (Figure 4a–c). During the experiment, other metabolites, such as hydroxylamine derivatives and amino derivatives generated by reducing the nitro group were also detected. Figure 4d shows the area under the curve of the relative amount of released 5′-DFUR. The amount of 5′-DFUR released from compound **2c** was significantly higher than those of **1c** and **3c**. Compounds **4c**, **5c** and **6c**, without nitro groups, remained well-stabilized under NTR conditions, with concentrations > 90% after 12 h. These results indicated that compound **2c** was the most suitable option since it featured a higher conversion and reaction rate.

#### 2.2.3. In Vitro Cytotoxicity Evaluation

The cytotoxicity of the target compounds **1c**–**6c** were evaluated in MCF-7 human breast cancer cells and HT29 human colon cancer cells. These two cell lines were chosen because 5′-DFUR is currently used to treat breast and gastrointestinal tumors. The cells were incubated with the target compounds at various concentrations for 24 h in the presence or absence of NTR. The cell viability and proliferation behavior were assessed by 3-(4,5-dimethylthiazol-2-yl)-2,5-diphenyltetrazolium (MTT) assay. The half-maximal inhibitory concentration values of the tested compounds for inhibiting cell proliferation are shown in Table 1. The cytotoxicity of compounds **1c**–**6c** was reduced by 4–8-fold compared to that of 5′-DFUR in the medium not containing NTR, suggesting that the structurally modified derivatives of 5′-DFUR have further reduced side effects in normal tissues compared to 5′-DFUR. The cytotoxicity of compouds **1c**–**3c** was significantly increased in the medium containing NTR (100 µg/mL), with that of compounds **2c** being comparable to that of 5′-DFUR. Compouns **4c**–**6c** showed no significant cytotoxicity in NTR-containing or -free media, suggesting that compouns **4c**–**6c**, which did not contain nitro moieties, were NTR-insensitive and could not be metabolized to produce 5′-DFUR. These results indicated that the nitro-containing moiety is key in the design of this class of prodrugs. Compound **2c** exhibited the highest selectivity ratio (4.99 in the MCF-7 cell line, 7.39 in the HT29 cell line) and was selected for further research.

#### 2.2.4. In Vivo Antitumor Activity Evaluation

**Figure 5:**
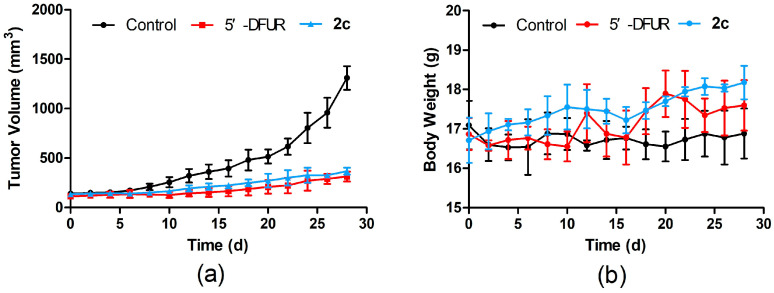
Antitumor activity of compound **2c** and 5′-DFUR in vivo. (**a**) Tumor volume over time in mice in the indicated groups; (**b**) body weight over time of mice in the indicated groups. Data are presented as mean  ±  standard deviation (*n* = 6). 5′-DFUR, 5′-deoxy-5-fluorouridine.

Compound **2c**, which has excellent stability, a high metabolic rate, and strong cytotoxicity under NTR conditions in vitro, was selected for the in vivo evaluation of antitumor activity in tumor-bearing mice. Tumor-bearing mice were generated by the axillary injection of HT29 colon cancer cells into C57BL/6 mice. When the tumors grew to approximately 100–150 mm^3^, the mice were randomly divided into negative control (vehicle), compound **2c** (50 mg/kg), and positive control 5′-DFUR (50 mg/kg) groups (*n* = 6 each). The mice were injected intraperitoneally every 2 days for 4 weeks, and the tumor volume and body weight of the mice were measured every 2 days. No death occurred in any group of mice during the experimental period. Compound **2c** exhibited a significant tumor growth inhibition (TGI) rate in vivo, with an effect comparable to that of 5′-DFUR (77.5% and 83.0% respectively) (Figure 5a). The body weights of the mice in the compound **2c** group showed smaller weight fluctuations and were slightly higher than those of the positive control group (Figure 5b). No significant drug-related toxic reactions were observed, suggesting that compound **2c** induced fewer side effects.

## 3. Materials and Methods

### 3.1. Chemistry

All commercially available materials and reagents were used without purification unless otherwise indicated. Purification was performed using silica gel (200–300 mesh, Qingdao Marine Chemical Company, Qingdao, China) and column chromatography. The purity and characterization of the compounds were established using HPLC (Agilent Technologies, Santa Clara, CA, USA) at a purity of >95% for all target compounds. NMR spectra (500 MHz for ^1^H NMR, 125 MHz for ^13^C NMR) were recorded on a Bruker AVANCE NEO 500 instrument (Bruker Biospin GmbH, Ettlingen, Germany) and analyzed in deuterated DMSO-*d*_6_ (Energy Chemical, Shanghai, China). Chemical shifts are reported in parts per million relative to tetramethylsilane (0.00 ppm) or solvent peaks as an internal reference. Splitting patterns are indicated as follows: s, singlet; d, doublet; t, triplet; m, multiplet; coupling constants (*J* values) are given in hertz. High-resolution mass spectrometry was conducted on a UPLC G2-XS QTOF spectrometer (Waters Corporation, Milford, MA, USA) using the electrospray ionization Fourier transform ion cyclotron resonance technique. The NMR, HRMS, and HPLC spectra of compounds **1c**–**6c** are presented in Supporting Information.

#### 3.1.1. General Synthetic Procedure for Bromide Intermediates **1b**–**6b**

Phosphorus tribromide (5.0 mmol; 1.0 equiv) dissolved in dichloromethane (5 mL) was slowly added to a solution of compounds **1a**–**6a** (5.0 mmol; 1.0 equiv) in dichloromethane (30 mL) at 0 °C. After stirring at 0 °C for 1 h, the reaction mixture was quenched with cold water (20 mL) and stirred at room temperature for 2 h. The mixture was extracted with dichloromethane (3 × 20 mL), and the organic phases were combined. The organic phases were washed with water (2 × 30 mL) and saturated brine (2 × 30 mL) before drying over anhydrous sodium sulfate. After filtration and concentration under reduced pressure, the residue was purified using a mixture of petroleum ether and ethyl acetate as the eluent to obtain the corresponding bromide intermediates **1b**–**6b**.

#### 3.1.2. General Synthetic Procedure for Target Compounds **1c**–**6c**

K_2_CO_3_ (2.0 mmol; 2.0 equiv) and a catalytic amount of KI (0.1 mmol; 0.1 equiv) was added to a solution of 5′-DFUR (1.0 mmol; 1.0 equiv) and compounds **1b**–**6b** (1.0 mmol; 1.0 equiv) in *N*,*N*-dimethylformamide (20 mL) at room temperature. The reaction mixture was stirred at room temperature for 12 h, and concentrated under reduced pressure after the reaction was complete (thin-layer chromatography). The residue was solubilized in ethyl acetate, washed with water (2 × 30 mL) and saturated brine (2 × 30 mL), and dried over anhydrous sodium sulfate. After filtration and concentration under reduced pressure, the residue was purified using a mixture of petroleum ether and ethyl acetate as the eluent to obtain the target products **1c**–**6c**.

### 3.2. Biological Evaluation

#### 3.2.1. Stability Evaluation of Target Compounds in PBS Versus Plasma

PBS (pH 7.4) was made with 137 mM NaCl, 2.7 mM KCl, 10 mM Na_2_HPO_4_, and 1.8 mM KH_2_PO_4_, while PBS (pH 6.5) was made with 24.6 mM Na_2_HPO_4_ and 175.4 mM NaH_2_PO_4_. The target compounds were dissolved into the different buffers (50 μM; 1% DMSO), and the mixtures were incubated at 37 °C. A 100-μL sample was transferred to a vial for HPLC analysis at 1, 2, 4, 8, 12, and 24 h. C57BL/6 mice and SD rat orbital whole blood was collected into anticoagulation tubes and centrifuged at 4000× *g* for 5 min at 4 °C to obtain plasma. The target compounds were dissolved into the plasma (50 μM; 1% DMSO) and incubated at 37 °C. Each sample (100 μL) was collected and precipitated using 100 μL of acetonitrile. The mixture was vortexed for 5 min and centrifuged at 10,000× *g* for 10 min. The supernatant was transferred to a vial for HPLC analysis after 1, 2, 4, 8, 12, and 24 h.

#### 3.2.2. In Vitro Release Evaluation of Target Compounds

The target compounds NTR and NADH were dissolved in 10 mM PBS (pH 7.4; 1% DMSO). The final concentrations of the target compounds NTR and NADH in the mixtures were 50 μM, 50 μg/mL, and 2 mM, respectively. The mixture were incubated at 37 °C. The sample (100 μL) was collected and quenched by 100 µL of cold methanol. The mixture was vortexed for 5 min and centrifuged at 10,000× *g* for 10 min. The supernatant was transferred to a vial for HPLC analysis at 0.25, 0.5, 1, 2, 4, 8, and 12 h.

#### 3.2.3. In Vitro Cytotoxicity Evaluation

MCF-7 human breast cancer cells or HT29 human colon cancer cells purchased from the National Collection of Authenticated Cell Cultures were cultured in RPMI 1640 medium, and McCoy’s 5A medium supplemented with 10% fetal bovine serum and 1% penicillin-streptomycin in in a humidified 5% CO_2_ incubator at 37 °C. Cells in the logarithmic growth phase were inoculated into 96-well plates at a density of 1.0 × 10^4^ cells per well and cultured for 24 h. The target compounds were dissolved in DMSO and diluted in medium to different concentrations. The cells were then incubated with different concentrations of the target compound for 24 h. The cells in the NTR treatment group were incubated with various concentrations of compounds, NTR (final concentration, 50 µg/mL) and NADH (final concentration, 2 mM) for 24 h. Cell viability and proliferation were measured by MTT assay. Cell viability was calculated according to the following formula: cell viability (%) = (OD value of treatment group–OD value of blank group)/(OD value of control group–OD value of blank group) × 100. The half-maximal inhibitory concentration values were calculated from concentration-response curves using GraphPad PRISM (version 5.0, GraphPad, San Diego, CA, USA).

#### 3.2.4. In Vivo Antitumor Activity Evaluation

Four-week-old C57BL/6 mice were purchased from Jinan Pengyue Experimental Animal Breeding Co. Ltd. (Jinan, China). The mice were housed in cages under a 12-h light/dark cycle from 7:00 to 19:00 at controlled temperatures (25–26 °C) and relative humidity (50 ± 10%) throughout the experimental period. All animals were allowed to eat and drink freely unless otherwise stated and allowed to acclimate for 1 week before the experiment. All animal experimental protocols were performed in accordance with applicable institutional and governmental regulations concerning the ethical use of animals (AP2024022954).

The tumor-bearing mice model was established by the subcutaneous injection of HT29 cells (1 × 10^7^ cells/mL) purchased from the National Collection of Authenticated Cell Cultures to the right axilla of C57BL/6 mice (100 µL/mouse). When the tumor size reached 100–150 mm^3^, the mice were evenly divided into negative control (vehicle), group **2c** (50 mg/kg), and 5′-DFUR (50 mg/kg) groups (*n* = 6 each). Compound **2c** and 5′-DFUR were dissolved in a solution containing 10% ethanol, 10% castor oil, and 80% saline that also served as the negative control. The mice were injected intraperitoneally every 2 days for 4 weeks, and the tumor volumes and body weights of the mice were measured every 2 days. Tumor volume was calculated using the following formula: tumor volume = length (mm) × width (mm) × width (mm) × 0.5. The TGI rate was determined as follows: TGI = 1 − (treatment final volume − treatment initial volume)/(control final volume − control initial volume) × 100%.

## 4. Conclusions

Conventional antitumor drugs have limited clinical applications because of their severe side effects. 5′-DFUR, a 5-FU prodrug, reduced the side effects caused by the use of 5-FU. However, 5′-DFUR also features side effects such as gastrointestinal reactions and liver and kidney injury. In this study, to reduce the side effects of 5′-DFUR, a novel series of 5′-DFUR derivatives was designed by introducing nitro-containing moieties into the structure of 5′-DFUR based on the high NTR levels in the tumor hypoxic microenvironment. Upon administration, 5-FU is generated under conditions of high NTR and TP in tumor tissues, which exerts antitumor activity and reduces toxic side effects in normal tissues.

The designed compounds were chemically synthesized, and their stability, release, cytotoxicity in vitro, and antitumor activity in vivo were examined. All compounds exhibited considerable stability in PBS and plasma at different pH values. Among them, compound **2c**, featuring nitrofuran fragments, was rapidly reduced in the presence of NTR and showed excellent hypoxic selectivity in the MCF-7 and HT29 cell lines. In addition, compound **2c** exhibited antitumor effects comparable to those of 5′-DFUR in vivo without significant toxic side effects. These results suggest that compound **2c** is an antitumor prodrug worthy of intensive research.

## Figures and Tables

**Table 1:** In vitro cytotoxicity assay of compounds **1c**–**6c** ^a^.

Compound	MCF-7			HT29		
	IC_50_ (µM)		IC_50_ (NTR-Free)/ IC_50_ (NTR)	IC_50_ (µM)		IC_50_ (NTR-Free)/ IC_50_ (NTR)
	NTR-Free	NTR		NTR-Free	NTR	
5′-DFUR	83.61	86.73	0.96	50.37	48.11	1.05
**1c**	469.85	106.27	4.42	583.74	78.96	7.39
**2c**	400.36	80.26	4.99	436.86	57.36	7.62
**3c**	435.26	96.35	4.52	456.93	63.28	7.22
**4c**	495.38	536.54	0.92	621.08	614.27	1.01
**5c**	412.35	423.24	0.97	459.36	421.63	1.09
**6c**	467.83	472.61	0.99	473.62	465.28	1.02

^a^ values are means of at least three experiments; 5′-DFUR, 5′-deoxy-5-fluorouridine; IC_50_, half-maximal inhibitory concentration; NTR, nitroreductase.

## Data Availability

The data presented in this study are available on request from the corresponding author.

## Supplementary Materials

The following supporting information can be downloaded at: https://www.mdpi.com/article/10.3390/molecules29215077/s1, Figure S1: Stability of compounds **4c**–**6c** in different pH PBS buffer and plasma; Figures S2–S25: Copies of NMR, HRMS and HPLC spectra of compounds **1c**–**6c**.
