# Supplementary material for: Design, Synthesis, and Evaluation of Doxifluridine Derivatives as Nitroreductase-Responsive Anticancer Prodrugs
Source: Molecules. 2024 Oct 27;29(21):5077. doi: 10.3390/molecules29215077 (PMC11547703; doi:10.3390/molecules29215077)
Supplement: Supplementary file 1 [file molecules-29-05077-s001.zip › molecules-3242413-supplementary.pdf]

Design, Synthesis, and Evaluation of Doxifluridine Derivatives as Nitroreductase-Responsive Anticancer Prodrugs

Xinmeng Zhang, Taimin Dong, Xu Li, Changjie Xu, Fanghui, Chen, Shiben Wang* and Xuekun Wang*

National Key Laboratory of Macromolecular Drug Development and Manufacturing, School of Pharmaceutical Sciences and Food Engineering, Liaocheng University, 1 Hunan Street, Liaocheng 252059, China; 18241016451@163.com (X.Z.); 17616455575@163.com (T.D.); lx221024@126.com (X.L.); 3494882434@qq.com (X.Z.); chenfanghui@lcu.edu.cn (F.C.)

* Correspondence: wangxuekun@lcu.edu.cn (X.W.); wangshiben@lcu.edu.cn (S.W.)

Stability of compounds **4c–6c** in different buffer and plasma was shown in Figure S1.

The NMR spectra (500 MHz for ^{1}H NMR and 125 MHz for ^{13}C NMR spectra) were recorded using a Bruker AVANCE NEO 500 instrument (compounds were dissolved in DMSO-d_6). Chemical shifts are shown as values relative to the internal standard (tetramethylsilane), and coupling constants (*J* values) are given in hertz (Hz). HRMS was conducted using a UPLC G2-XS Qtof spectrometer (Waters) with the electrospray ionization Fourier transform ion cyclotron resonance (ESI-FTICR) technique. Agilent 1260 Infinity HPLC system (Agilent Technologies) was used for HPLC analyses.

1. Stability of compounds 4c–6c in different buffer and plasma

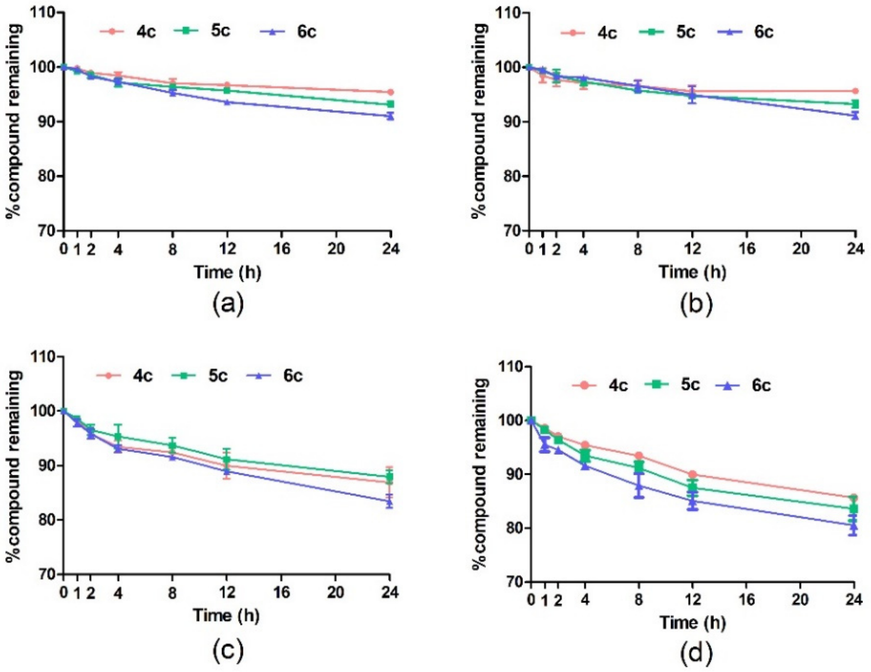

Figure S1. Stability of compounds **4c–6c** in different buffer and plasma: (A) pH = 6.5 PBS; (B) pH = 7.4 PBS; (C) mouse plasma; (D) rat plasma.

2. The NMR, HRMS and HPLC spectra of compounds 1C–6C

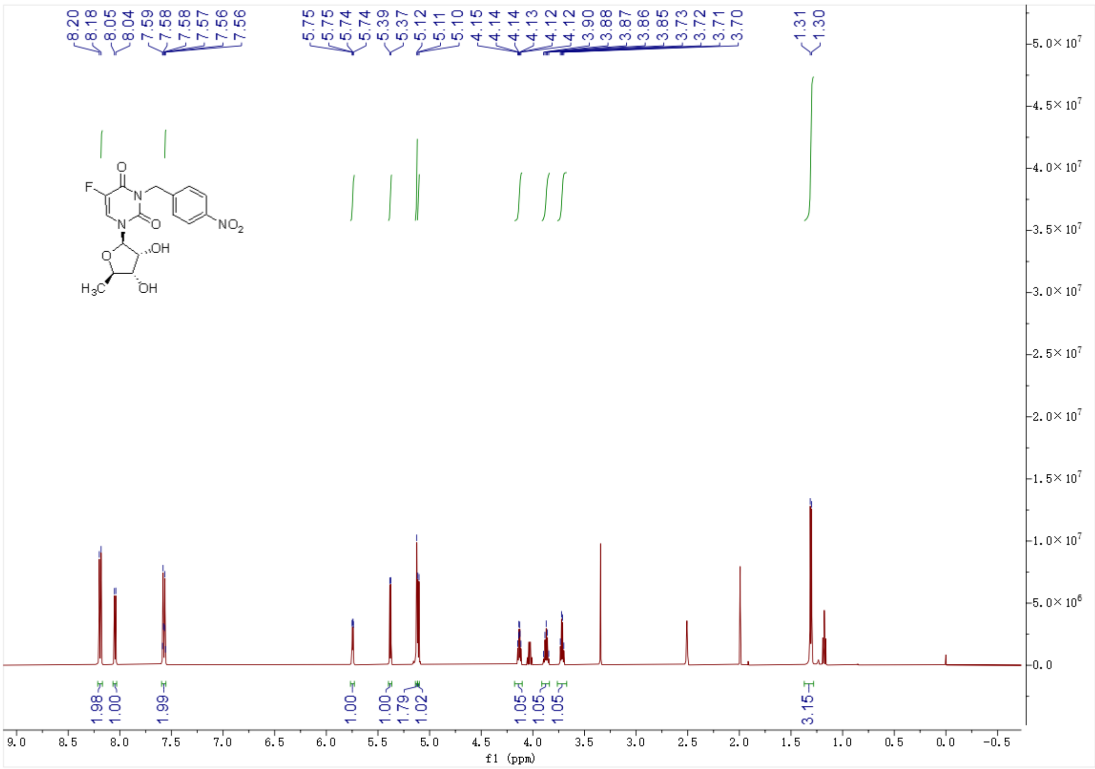

Figure S2. ^{1}H NMR spectrum for **1C** (DMSO-*d6*, 500 MHz)

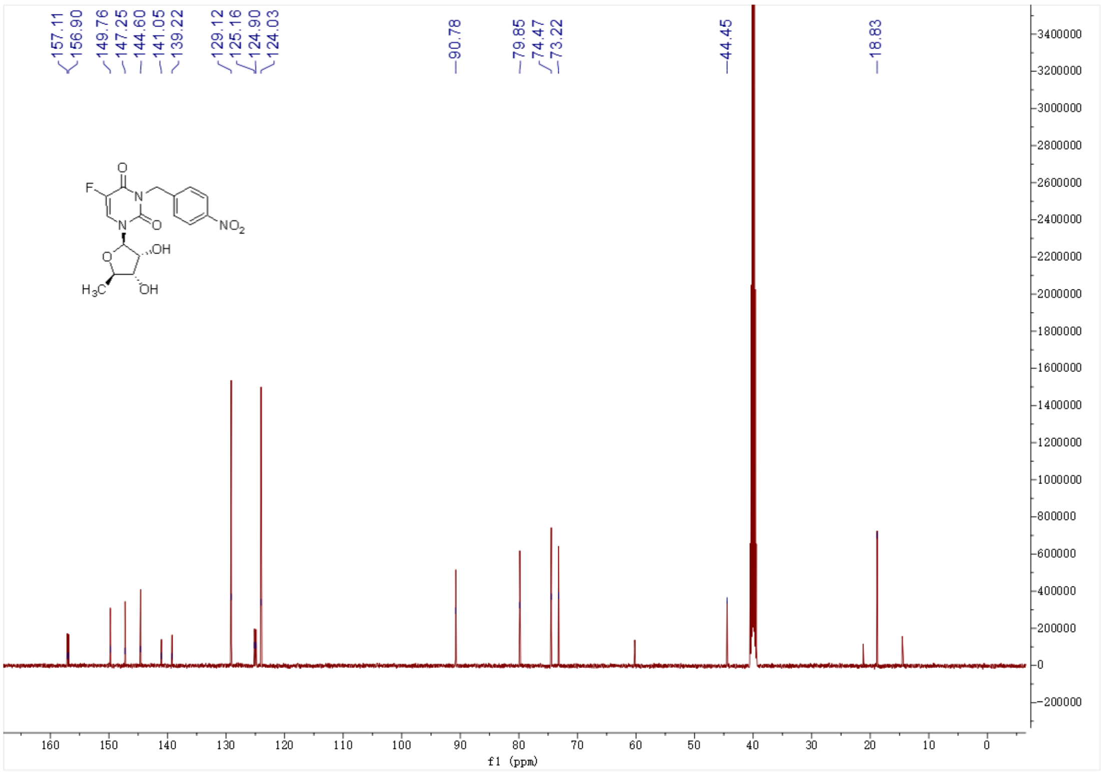

Figure S3. ^{13}C NMR spectrum for **1C** (DMSO-*d6*, 125 MHz)

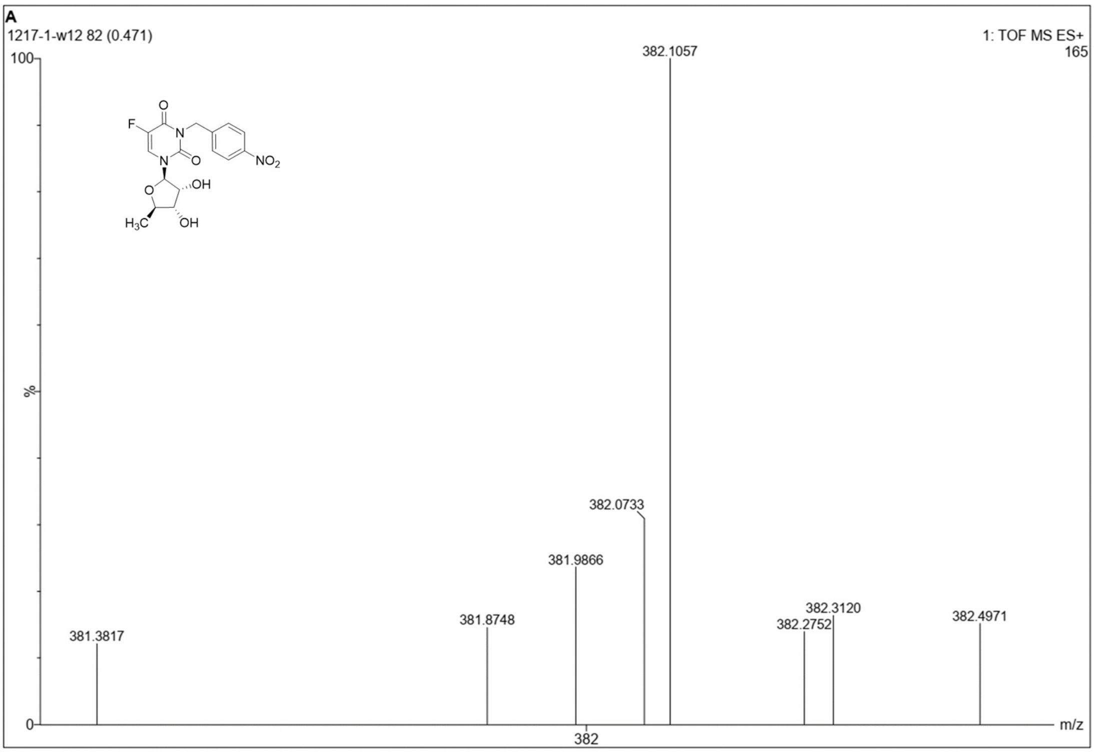

Figure S4. HRMS spectra of compound **1c**

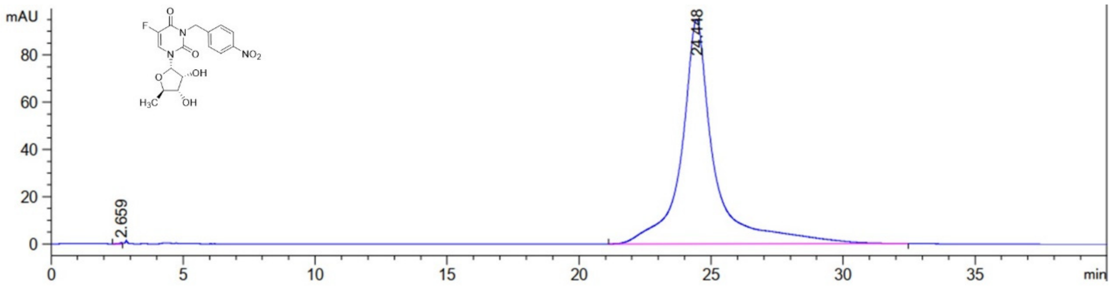

Peak #	Retention time [min]	Type	Peak width [min]	Peak area [mAU*s]	Peak height [mAU]	Peak area ratio %
1	2.754	VV	0.0685	2.60358	5.57812e-1	0.0402
2	24.448	BB	1.2033	6472.83301	74.08953	99.9598

Figure S5. HPLC spectra of compound **1c**

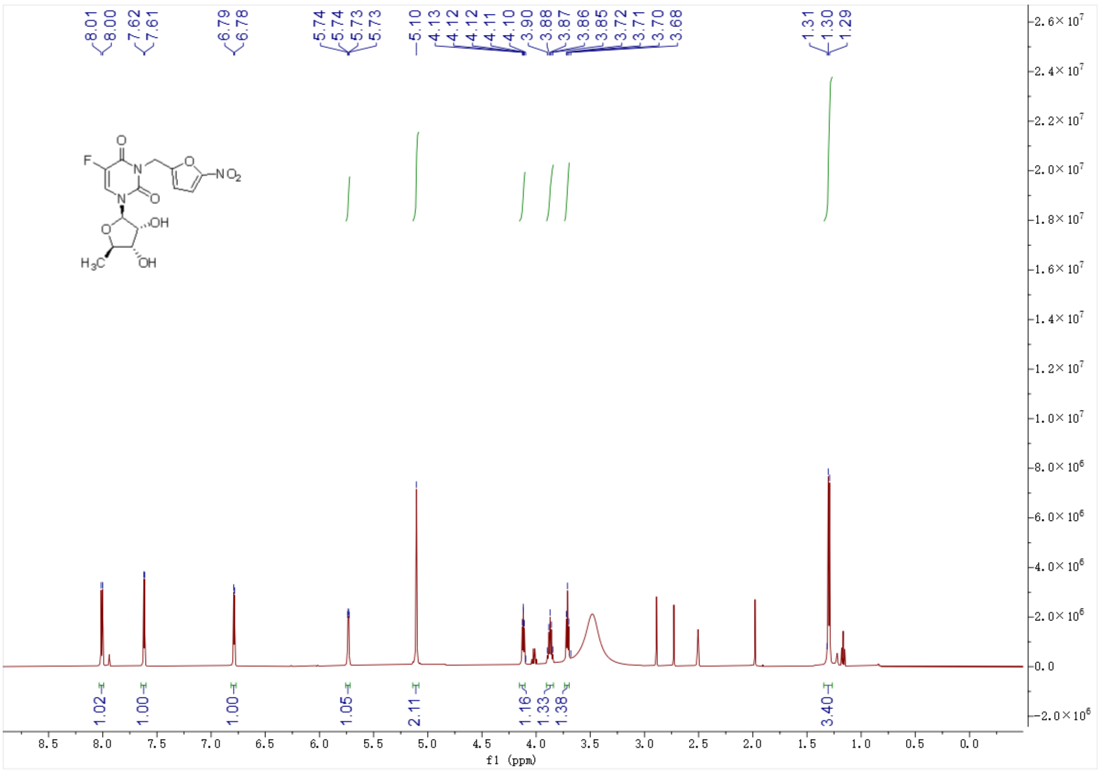

Figure S6. ^{1}H NMR spectrum for **2C** (DMSO-*d6*, 500 MHz)

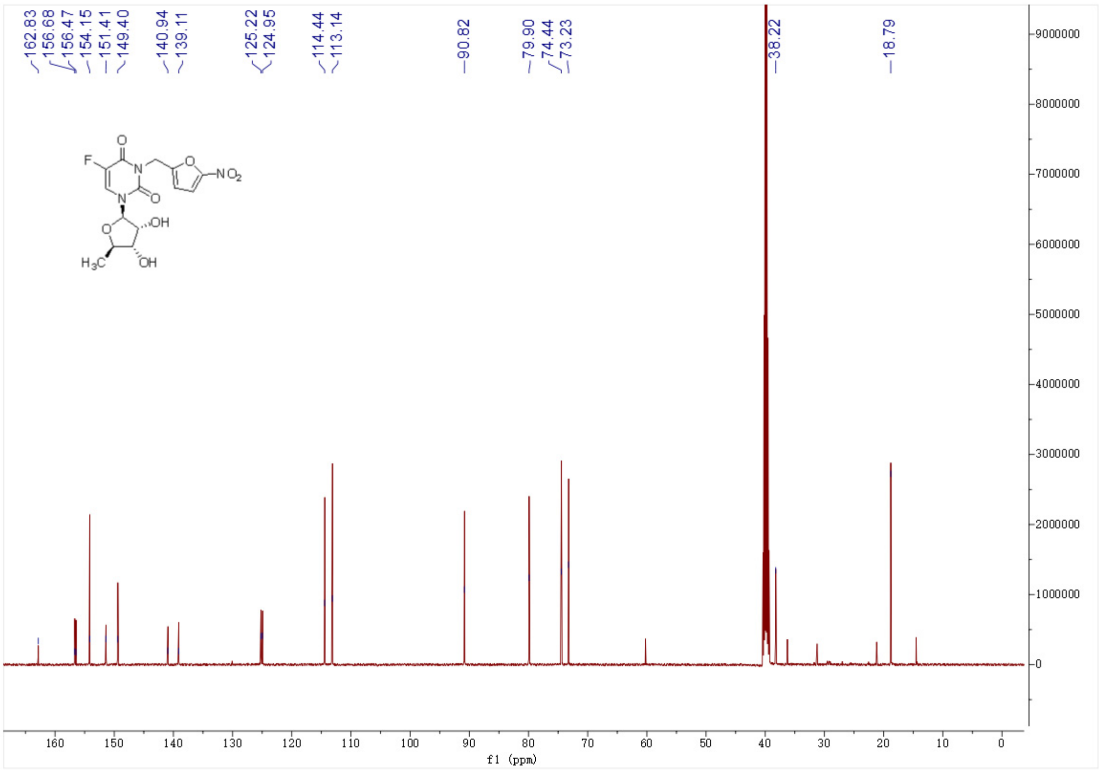

Figure S7. ^{13}C NMR spectrum for **2C** (DMSO-*d6*, 125 MHz)

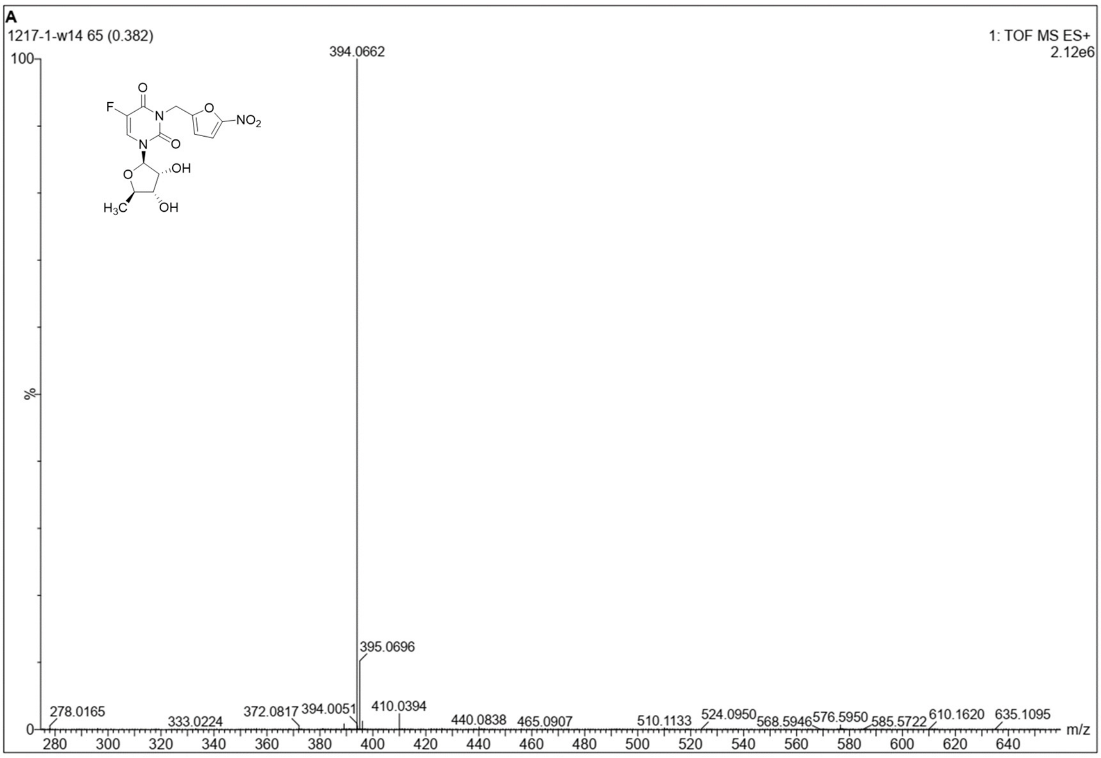

Figure S8. HRMS spectra of compound **2c**

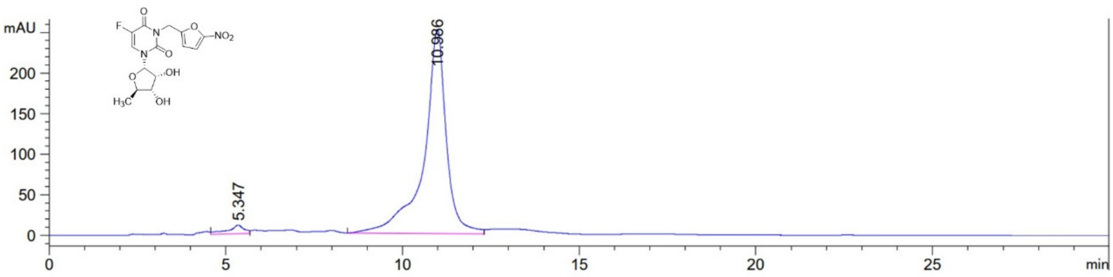

Peak #	Retention time [min]	Type	Peak width [min]	Peak area [mAU*s]	Peak height [mAU]	Peak area ratio %
1	5.347	VV	0.4296	336.07831	10.49512	2.9484
2	10.986	BV	0.6168	1.10625e4	251.66934	97.0516

Figure S9. HPLC spectra of compound **2c**

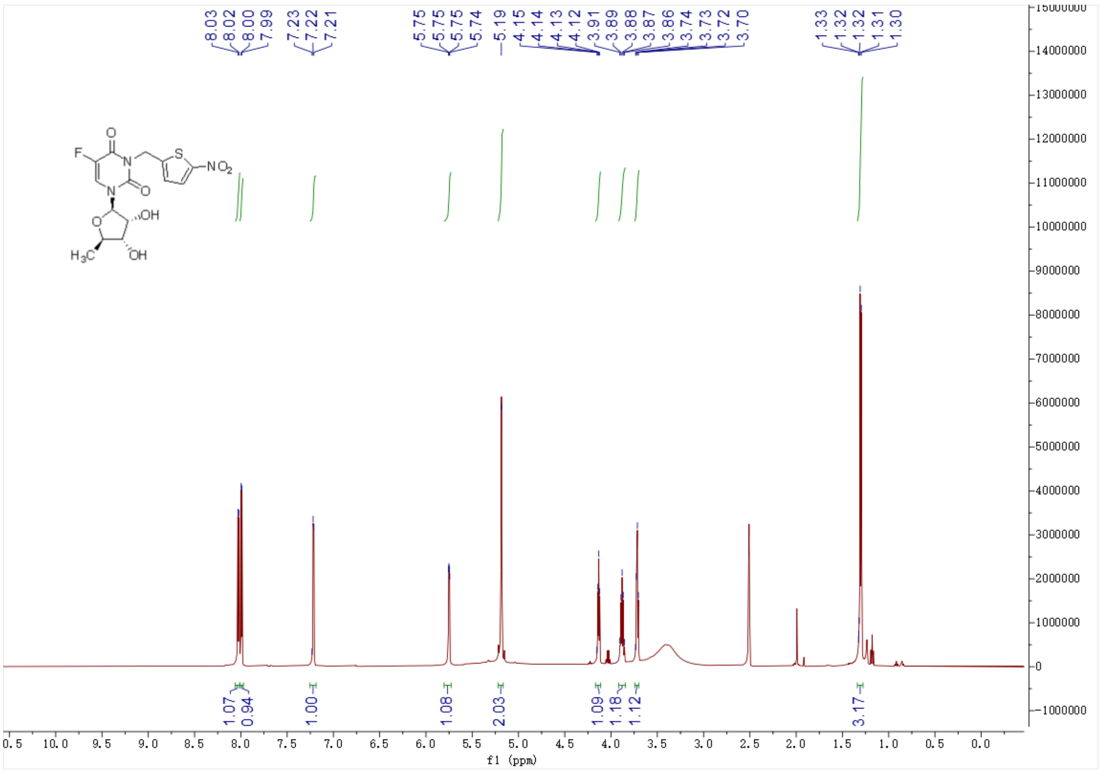

Figure S10. ^{1}H NMR spectrum for **3C** (DMSO-*d6*, 500 MHz)

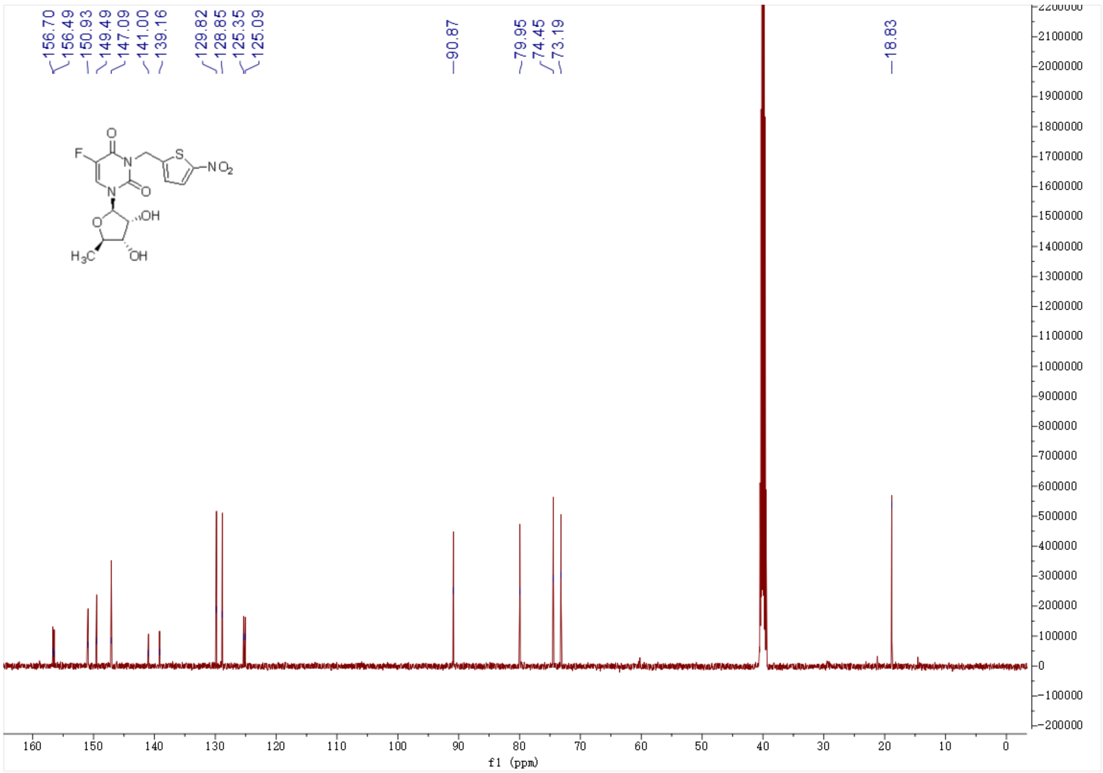

Figure S11. ^{13}C NMR spectrum for **3C** (DMSO-*d6*, 125 MHz)

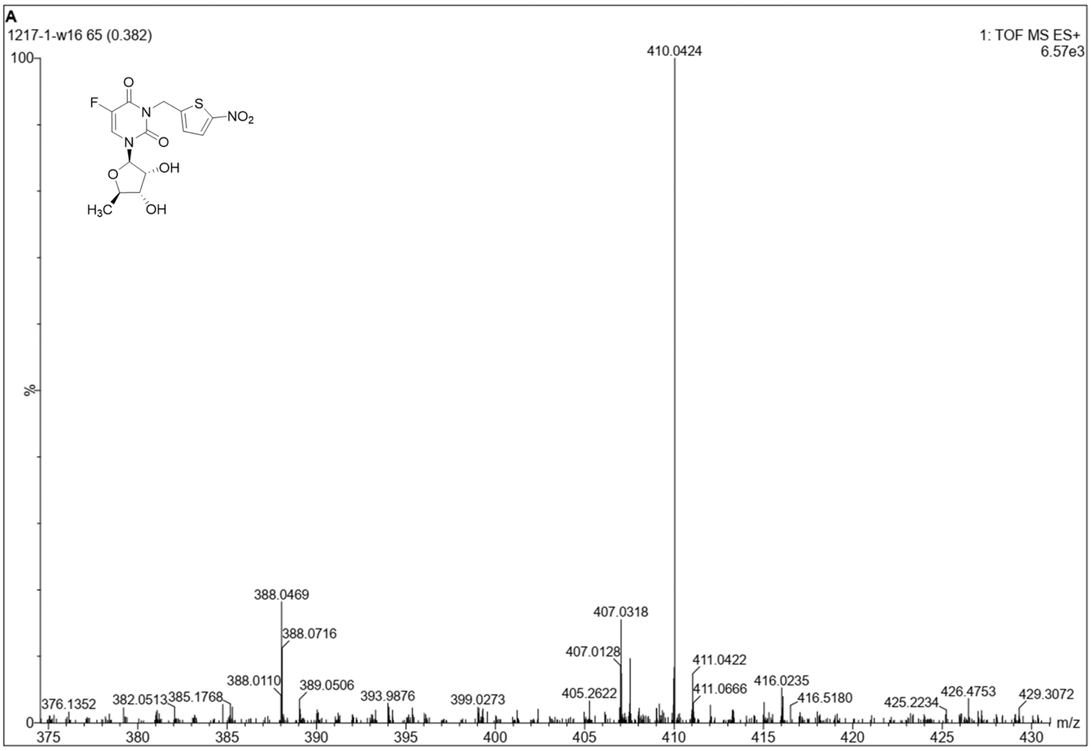

Figure S12. HRMS spectra of compound **3c**

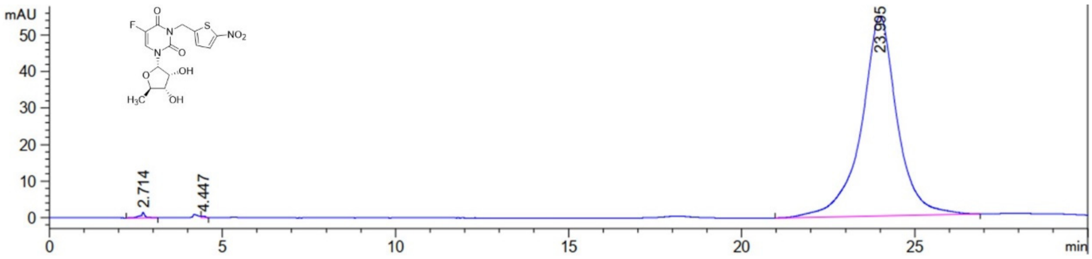

Peak #	Retention time [min]	Type	Peak width [min]	Peak area [mAU*s]	Peak height [mAU]	Peak area ratio %
1	2.714	BB	0.1231	13.80630	1.50388	0.3492
2	4.447	VB	0.1085	3.12583	4.31325e-1	0.0791
3	23.995	BB	1.0383	3936.26978	54.78447	99.5717

Figure S13. HPLC spectra of compound **3c**

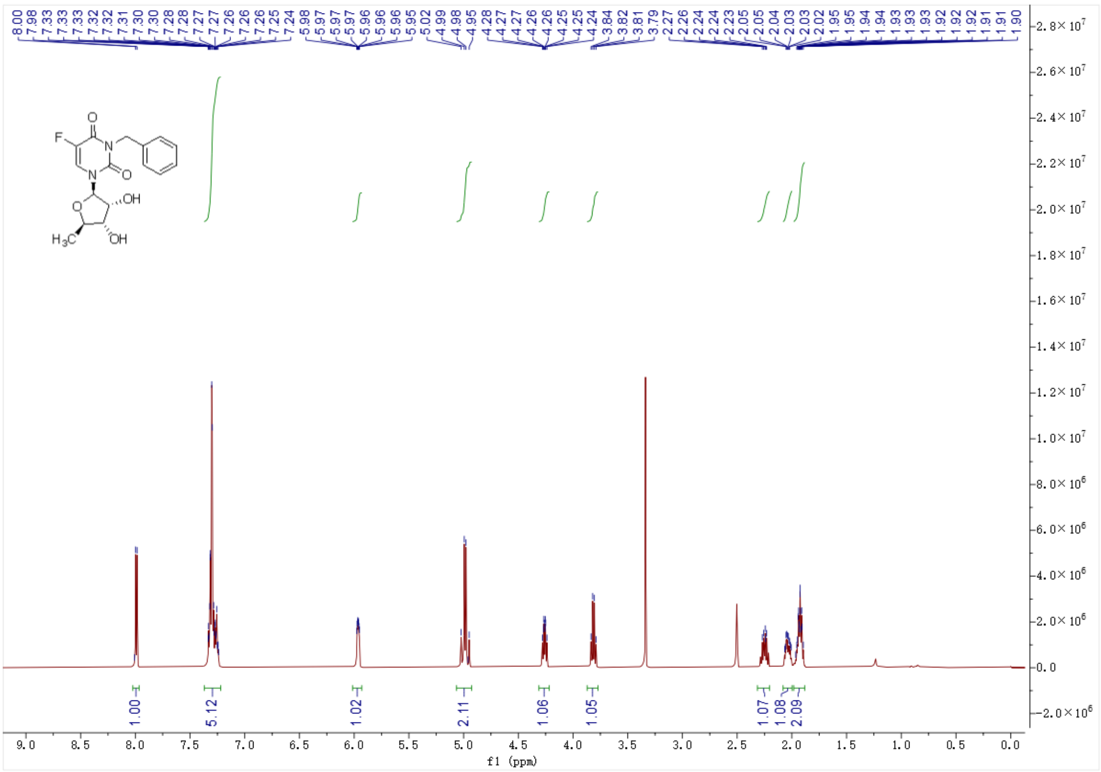

Figure S14. ^{1}H NMR spectrum for **4C** (DMSO-*d6*, 500 MHz)

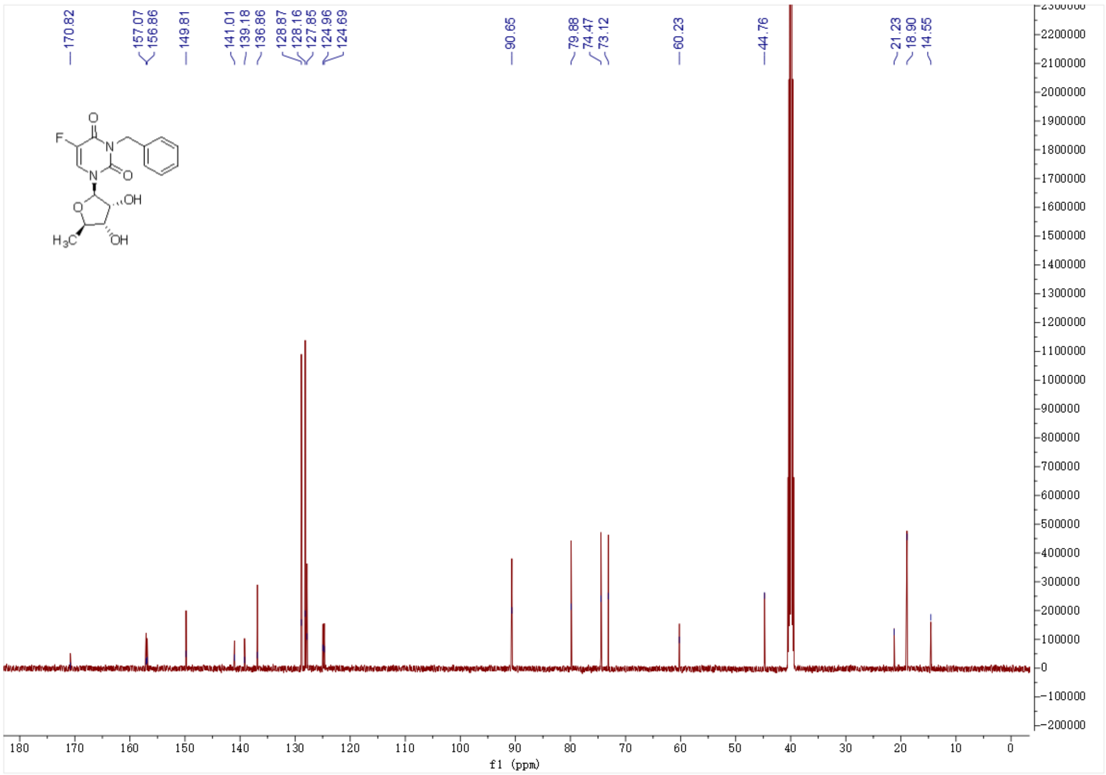

Figure S15. ^{13}C NMR spectrum for **4C** (DMSO-*d6*, 125 MHz)

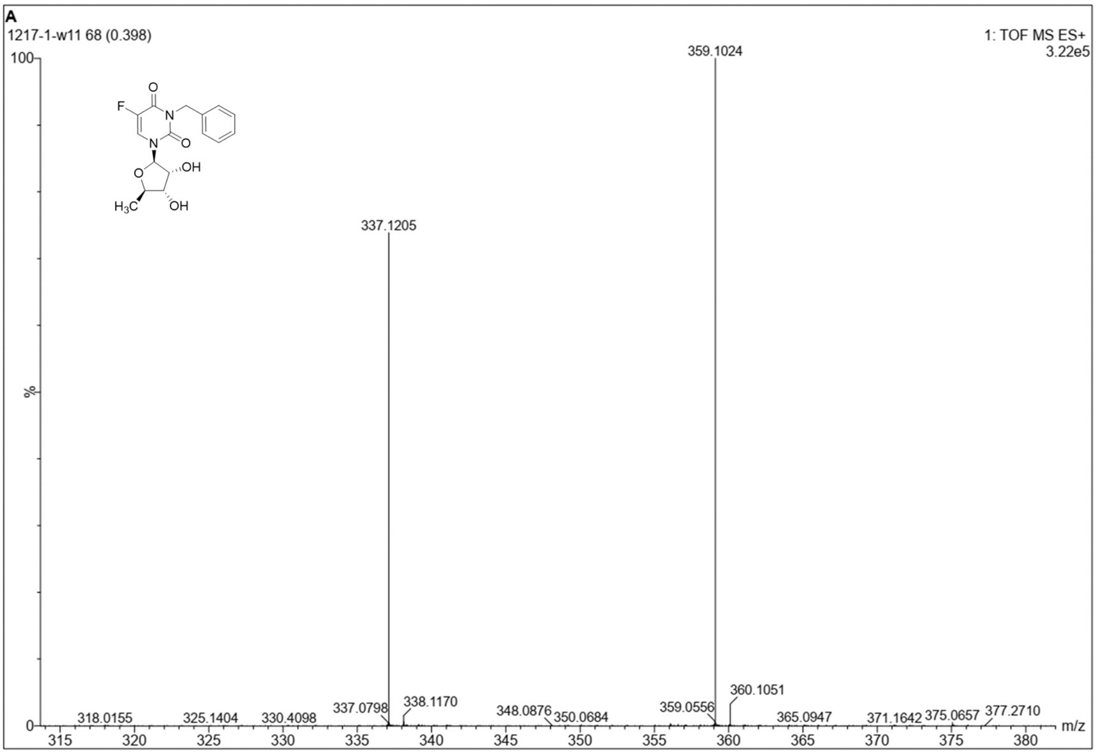

Figure S16. HRMS spectra of compound **4c**

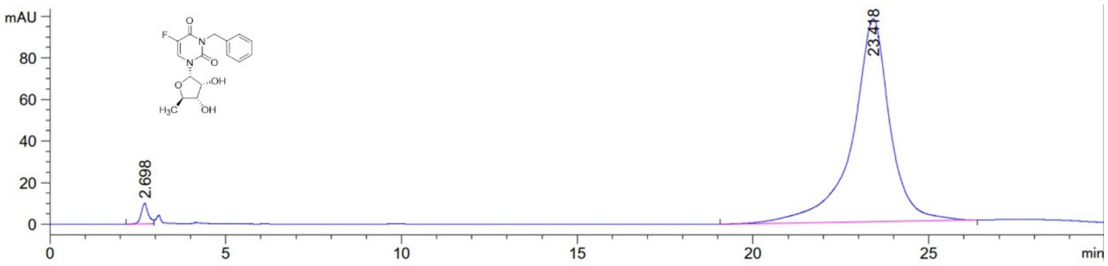

Peak #	Retention time [min]	Type	Peak width [min]	Peak area [mAU*s]	Peak height [mAU]	Peak area ratio %
1	2.698	BV	0.2128	141.69124	10.02818	1.9023
2	23.418	BB	1.0617	7306.66992	97.56462	98.0977

Figure S17. HPLC spectra of compound **4c**

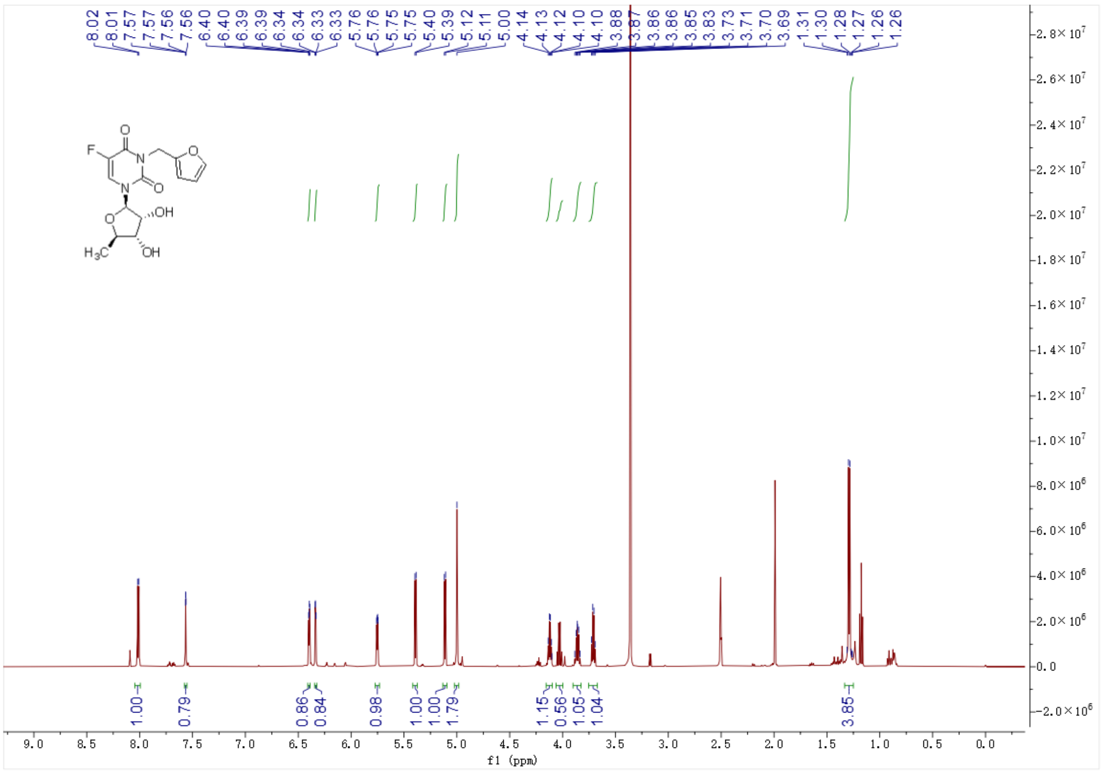

Figure S18. ^{1}H NMR spectrum for **5C** (DMSO-*d6*, 500 MHz)

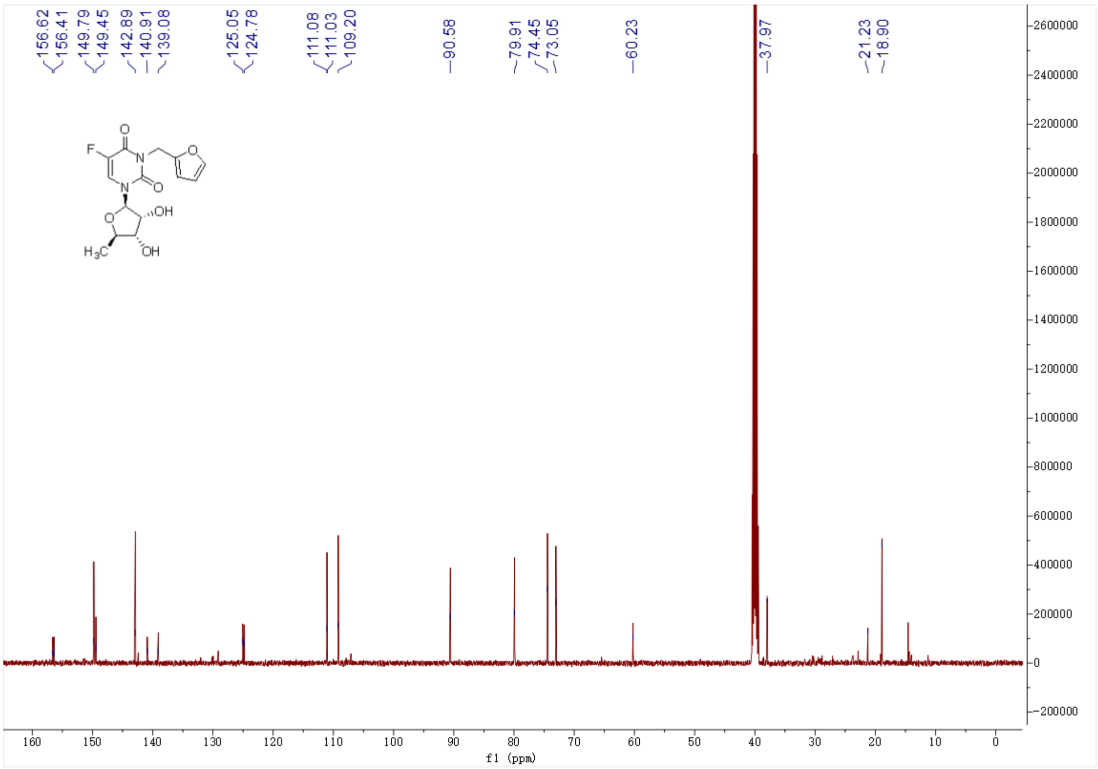

Figure S19. ^{13}C NMR spectrum for **5C** (DMSO-*d6*, 125 MHz)

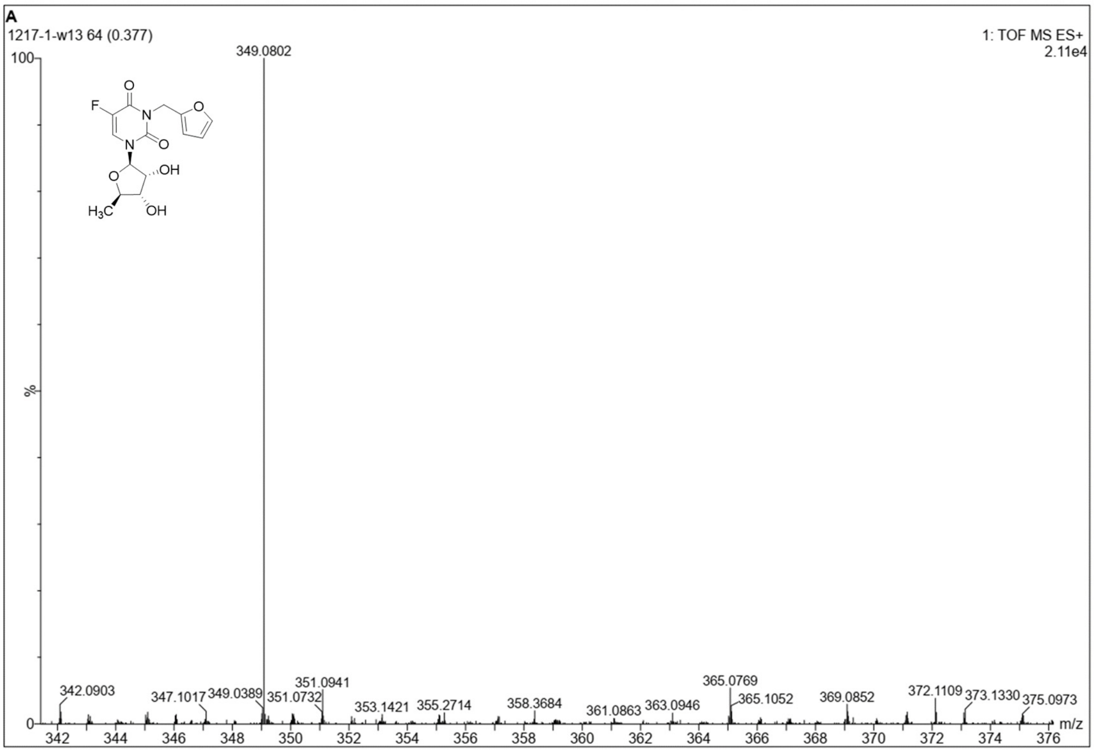

Figure S20. HRMS spectra of compound **5c**

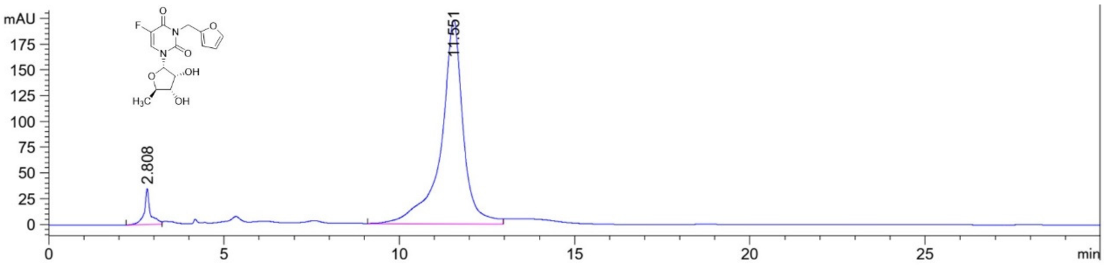

Peak #	Retention time [min]	Type	Peak width [min]	Peak area [mAU*s]	Peak height [mAU]	Peak area ratio %
1	2.808	BV	0.1683	431.96655	34.93167	4.7289
2	11.551	BV	0.6261	8702.63086	197.50623	95.2711

Figure S21. HPLC spectra of compound **5c**

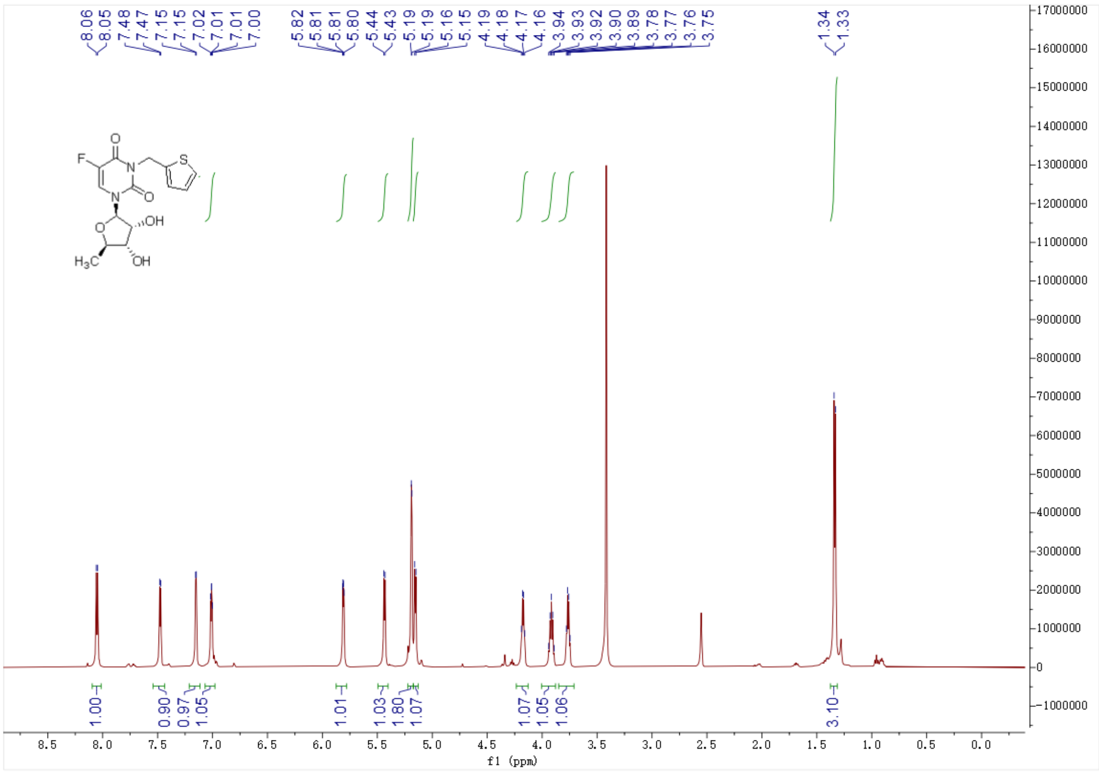

Figure S22. ^{1}H NMR spectrum for **6C** (DMSO-*d6*, 500 MHz)

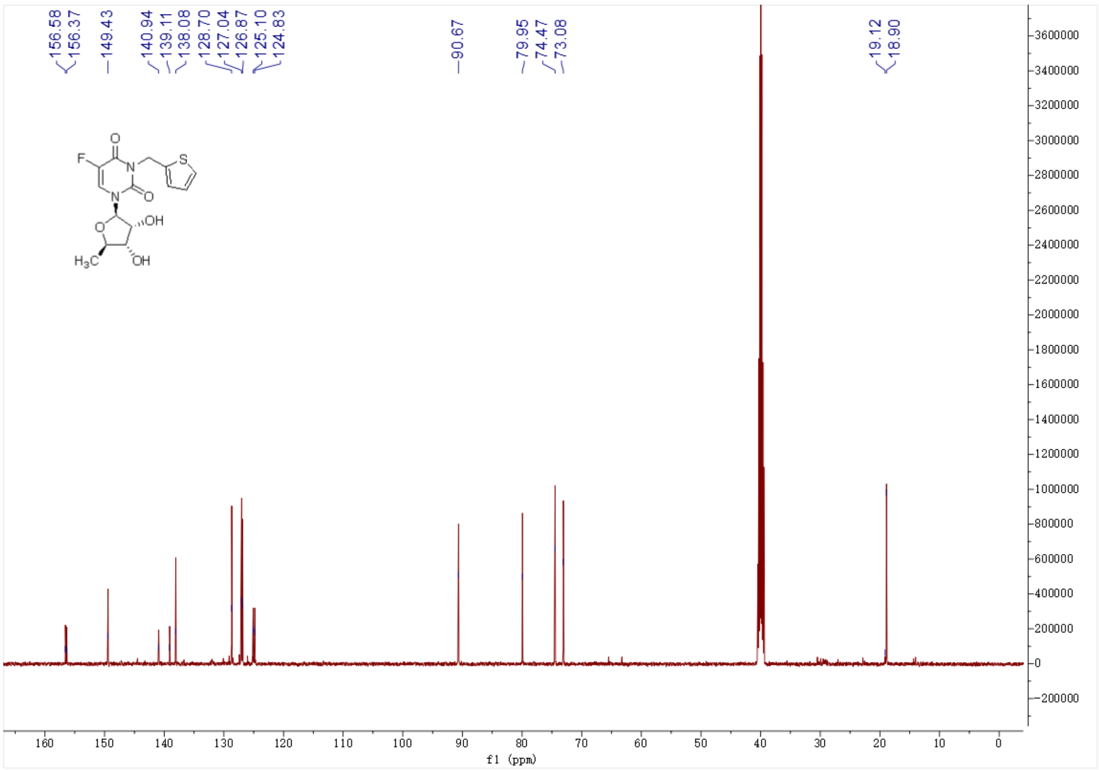

Figure S23. ^{13}C NMR spectrum for **6C** (DMSO-*d6*, 125 MHz)

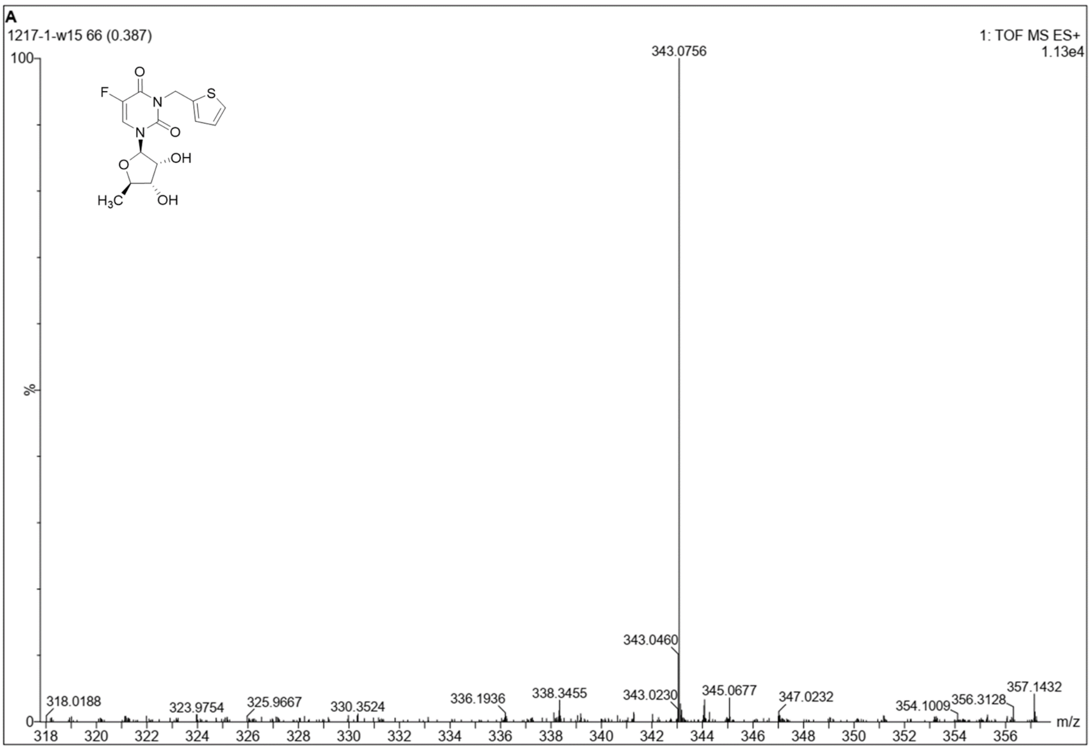

Figure S24. HRMS spectra of compound **6c**

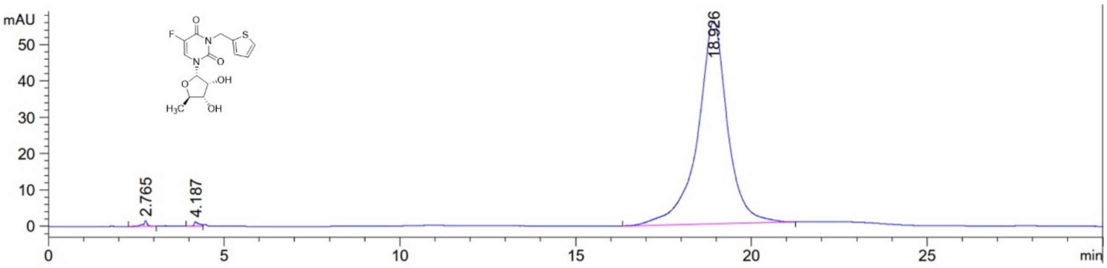

Peak #	Retention time [min]	Type	Peak width [min]	Peak area [mAU*s]	Peak height [mAU]	Peak area ratio %
1	2.765	BV	0.1135	13.26920	1.58837	0.3986
2	4.187	BV	0.1330	11.41527	1.15760	0.3429
3	18.926	BB	0.8547	3304.03833	55.80571	99.2584

Figure S25. HPLC spectra of compound **6c**
